# Rationale, challenges, and participants in a Phase II trial of a botanical product for chronic hepatitis C

**DOI:** 10.1177/1740774511427064

**Published:** 2012-02

**Authors:** K Rajender Reddy, Steven H Belle, Michael W Fried, Nezam Afdhal, Victor J Navarro, Roy L Hawke, Abdus S Wahed, Edward Doo, Catherine M Meyers, for the SyNCH Study Group

**Affiliations:** a University of Pennsylvania, Philadelphia, PA, USA; b University of Pittsburgh, Pittsburgh, PA, USA; c University of North Carolina, Chapel Hill, NC, USA; d Beth Israel Deaconess Medical Center, Boston, MA, USA; e Thomas Jefferson University, Philadelphia, PA, USA; f National Institutes of Health, National Institute of Diabetes and Digestive and Kidney Diseases, Bethesda, MD, USA; g National Institutes of Health, National Center for Complementary and Alternative Medicine, Bethesda, MD, USA

## Abstract

***Background*** Chronic hepatitis C is associated with significant morbidity and mortality as a consequence of progression to cirrhosis, hepatocellular carcinoma, and liver failure. Current treatment for chronic hepatitis C with pegylated interferon (IFN) and ribavirin is associated with suboptimal responses and numerous adverse effects. A number of botanical products have been used to treat hepatic disorders. Silymarin, extracted from the milk thistle plant, *Silybum marianum* (L) Gaertn. (Asteraceae), has been most widely used for various liver disorders, including chronic hepatitis C, B, and alcoholic liver disease. However, the safety and efficacy of silymarin have not been studied systematically in chronic hepatitis C.

***Purpose*** We describe our strategy for a phased approach for studying the impact of silymarin in hepatitis C, in the context of the unique challenges of botanical product clinical trials and the development of specific and curative antiviral therapy.

***Methods*** This multicenter, randomized, double-masked, placebo-controlled trial was conducted with four clinical centers and a data-coordinating center in the United States, to assess the impact of silymarin therapy in patients with chronic hepatitis C who failed conventional antiviral therapy.

***Results*** Key aspects relevant to performing clinical trials of botanical products include early identification of an appropriate product with standard product chemistry, acquisition of pharmacokinetic and dosing information, selection of the appropriate study group, and choosing rigorous outcome variables.

***Potential limitations*** Trial participants were chronic hepatitis C patients who were nonsustained virologic responders to IFN-based therapy; therefore, the findings are not generalizable to all hepatitis C populations. Further, alanine aminotransferase, a biochemical liver test, rather than hepatitis viral RNA or liver histology was the primary end point.

***Conclusions*** The challenges identified and addressed during development of this United States multicenter Phase II trial to evaluate silymarin for treatment of patients with chronic hepatitis C infection who had failed to respond successfully to previous IFN-based therapy are common and must be addressed to conduct rigorous trials of botanical products.

## Introduction

The hepatitis C virus (HCV) is a heterogeneous virus; it is estimated that 170 million people are infected worldwide [1]. The prevalence of antibodies to hepatitis C in the United States is approximately 1.6%, representing 4.1 million anti-HCV-positive persons, according to the National Health and Nutrition Examination Survey [1]. Chronic viral hepatitis infection can lead to cirrhosis, hepatic decompensation (i.e., liver failure), hepatocellular carcinoma (i.e., liver cancer), and death. Further, approximately 10,000 deaths result from hepatitis C-associated complications annually. End-stage liver disease due to chronic hepatitis C is the main indication for liver transplantation [2]. Current estimates suggest that there will be a continually increasing disease burden, at least over the next couple of decades, from hepatitis C and its complications [2].

While there have been some advances in the treatment for chronic hepatitis C infection, therapeutic options remain limited. Over the past two decades, treatment has evolved from standard interferon (IFN) monotherapy to current standard of care with pegylated IFN and ribavirin [3–5]. Apart from less than ideal response rates for most HCV genotype 1 patients in the United States, there is a more difficult issue of tolerance of therapy. Both IFN and ribavirin have numerous side effects that are frequently not well-tolerated by patients; several studies estimate that up to 86% of patients are deferred from conventional IFN treatment due to medical or psychiatric comorbidities [6–8]. Substantial numbers of patients with chronic HCV in the United States therefore do not receive available therapies or are nonresponders to current treatment regimens, due at least in part to the inability to tolerate full therapy [3–5]. With the dearth of treatment options for such a common and serious disease, it is not surprising that both clinicians and patients have an interest in studying other potential therapies for treatment-resistant chronic HCV infection that may have a better side effect profile.

Silymarin, extracted from the milk thistle plant, *Silybum marianum*, is one of a variety of botanical products that have been used to treat hepatic disorders, including chronic hepatitis C, B, and alcoholic liver disease [9–12]. Silymarin is primarily a mixture of at least six major isomeric flavonolignans and a few flavonoids and is touted for its antioxidant properties [13]. Data from the National Health Interview Survey (NHIS) from 2007 indicate that 20% of the U.S. population uses botanical products regularly, and that silymarin use constitutes nearly 5% of this product exposure [14].

## Limitations of current silymarin data

Although largely in vitro evidence has accumulated on silymarin and its putative antiviral, immunomodulatory, and antifibrotic properties, clinical studies have not suggested a clinical benefit for silymarin in HCV infection [9–11,15–21]. A review of clinical studies of botanical products, including silymarin, that have been used for hepatic diseases revealed a lack of consistency in botanical preparations, dosing, clinical outcome measures, and length of treatment. There was no firm evidence that botanical products conferred clinical benefit in HCV infection [9–11].

As is common with clinical trials of all or most botanical products, studies are limited by a lack of standardization of the product to be tested and lack of pharmacokinetic and dosing data. In addition, the outcome measures may not be comparable with those used in other studies. Furthermore, information on duration of treatment and follow-up are often lacking. For clinical trials of conventional pharmaceuticals, extensive preliminary preclinical and clinical studies (i.e., pharmacokinetics, dosing, and mechanistic studies) are undertaken early in product development, prior to studies testing efficacy, due to regulatory requirements of the U.S. Food and Drug Administration (FDA). Such data, however, typically are not required, or even available, for most marketed botanical products, including silymarin. Also, unlike conventional drugs, botanicals are typically mixtures of uncharacterized constituents; particular attention must be given to analyzing all potentially active ingredients. Therefore, it is essential to perform early-phase testing of botanical products to provide such critical information before embarking on large-scale clinical trials.

## Identifying the silymarin manufacturer

**Figure 1.**
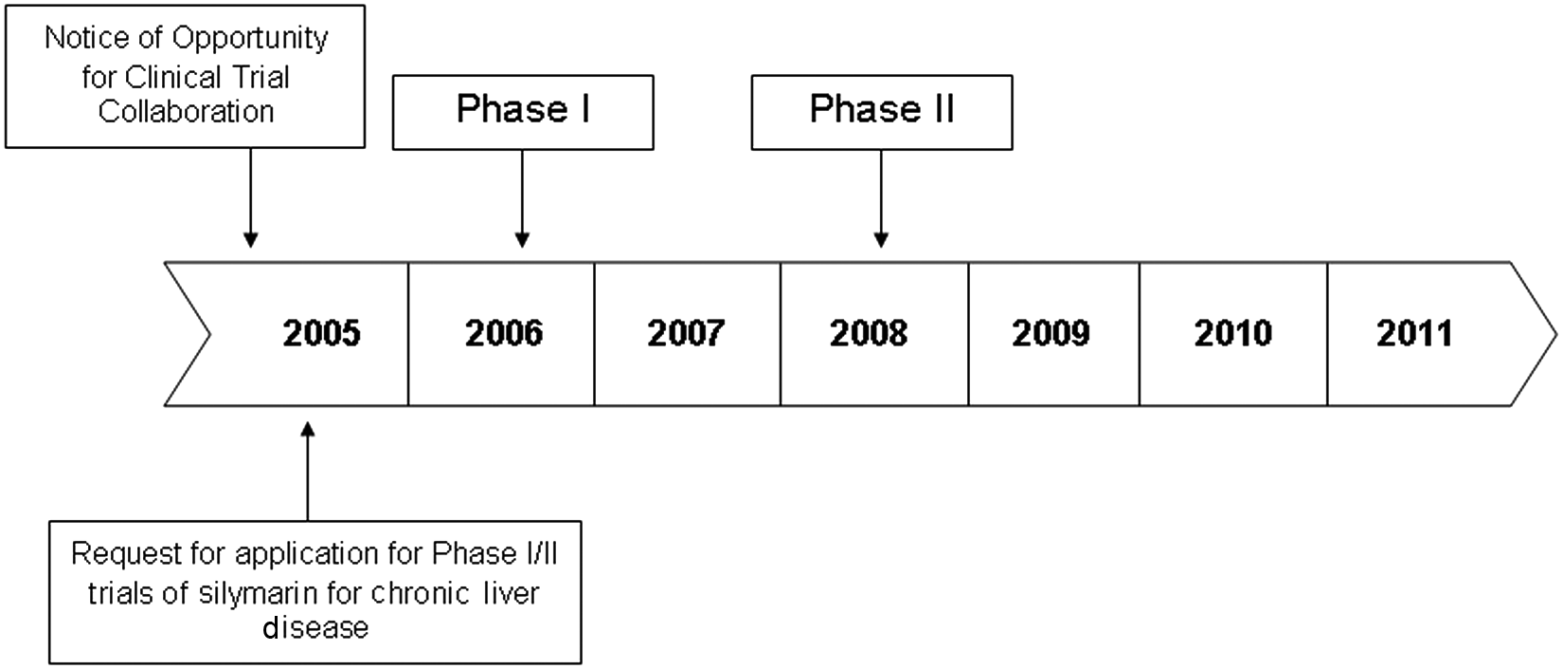
Chronology of planning and implementation of silymarin Phase I and Phase II studies.

Prior to issuing the request for applications (RFA) for the clinical trials of silymarin in 2005 (Figure 1), the U.S. National Institutes of Health (NIH), *via* the National Center for Complementary and Alternative Medicine (NCCAM), issued a Notice of Opportunity for Clinical Trial Collaboration to identify manufacturers of silymarin that would be interested in donating product and placebo for proposed future trials. The early identification of a manufacturer was essential in developing a clinical trial for this botanical product. Unlike conventional pharmaceuticals, there is considerable variability in composition and chemistry of marketed botanical products. Therefore, it was necessary to identify a manufacturer that could provide appropriate product information. Moreover, as dietary supplements are marketed in the United States without requirements for standard chemistry, manufacturing and controls, or providing preclinical data to the FDA, the manufacturer selected was expected to be willing to work with both NIH and FDA to provide such information for silymarin.

Silymarin manufacturers who responded to the Notice of Opportunity were requested to provide NIH/NCCAM information regarding their silymarin product as it was anticipated that a trial of silymarin for a chronic liver disease would require approval of an Investigational New Drug (IND) application from the FDA; thus, product specifications would have to comply with standard FDA requirements. NIH/NCCAM selected the manufacturer based on several components of the product information, including product chemistry, manufacturing and controls, preclinical data, clinical data, information on regulatory filings, and the ability to provide support for phased clinical trials. Following review of responses from the manufacturers, the Legalon^®^ 140 brand of silymarin marketed by Rottapharm|Madaus (Monza, Italy and Cologne, Germany) was selected for the silymarin trials.

## Study organization

After identifying the silymarin manufacturer, NIH/NCCAM issued an RFA in 2005 for Phase I/II trials of silymarin for chronic liver disease (Figure 1) in order to assemble a consortium (the Silymarin in Non-alcoholic Steatohepatitis and C Hepatitis (SyNCH) Study Group, Appendix). The National Institute of Diabetes and Digestive and Kidney Diseases (NIDDK) also participated in the project. This Phase II multicenter trial was conducted with four clinical centers and a data-coordinating center (DCC) in the United States. The steering committee for the consortium comprises the principal investigators from the clinical centers, one of whom is the steering committee chair, and the DCC and two NIH project scientists (NIH/NCCAM and NIH/NIDDK). The steering committee members designed and implemented the clinical trials of this consortium. A Data and Safety Monitoring Board (DSMB) was established by NIH/NCCAM to provide trial oversight.

### Investigational product

Legalon^®^ 140 is a milk thistle fruit extract (*Silybum marianum* (L) Gaertn. (Asteraceae)) standardized to 140 mg of silymarin per gelatin capsule (53% as total silibinin, quantified photometrically). A Legalon^®^ 140 capsule contains 180 mg of dried extract of milk thistle fruits, or 140 mg of silymarin, which is the presumed active ingredient, or 108 mg of silymarin quantified as silibinin by high-pressure liquid chromatography. The total weight of the capsule is 429 mg and includes other milk thistle components of the extraction process, excipients, and the capsule shell. The 108 mg of silymarin in Legalon^®^ capsules consists of the following potentially active ingredients: silibinins A + B (~50 mg), silicristin (~20 mg), silidianin (~15 mg), and isosilibinins A + B (~15 mg). The placebo gelatin capsules used in the trial were identically sized capsules filled with a powder containing lactose and other components of Legalon^®^ 140, including mannitol, magnesium stearate, and sodium carboxymethyl starch. The phased trials in this program were conducted under an IND, held by NIH/NCCAM, from the FDA.

## Phase I study and selection of doses for Phase II

In view of suggestive in vitro evidence, a phased-study approach was developed by the SyNCH Study Group (Figure 1) to study the potential role of silymarin in research participants with chronic hepatitis C who were previously refractory to conventional therapy. Since preliminary dosing and pharmacokinetic data on the product were not available, a Phase I dose-ranging study in research participants with chronic HCV was performed initially by the study team [22]. The research participants were randomized to one of four silymarin doses covering a fivefold range (140, 280, 560 and 700 mg, thrice daily), to assess safety and dose–exposure relationships in HCV patients who were previously treatment nonresponders. No drug-related adverse events were reported during the Phase I study, and daily oral doses of up to 2.1 g were well tolerated. Nonlinear pharmacokinetics of silibinins A and B were noted, with data suggesting that the otherwise low bioavailability of silymarin may be overcome at higher than usual doses (700 mg thrice daily). Since no adverse events were observed at the highest exposures obtained with a dose of 700 mg, this dose was identified as the high dose for Phase II trial, as it was expected to provide reliable exposures and to be safe and well tolerated in the HCV population. In addition, the dose burden of five pills three times daily was considered the largest at which acceptable patient compliance can be expected. As dose–exposure proportionality was observed over the dose range of 140–700 mg of silymarin, a dose of 420 mg was selected for the lower dose in the Phase II trial, as it would be expected to result in 40% lower exposure than the 700-mg dose, which might be associated with clinically significant differences in pharmacodynamic end points.

The Phase II study, the subject of this report, was a placebo-controlled, double-masked, randomized trial that focused on safety and efficacy of silymarin at two distinct doses (420 and 700 mg, thrice daily) for treating patients with chronic HCV infection who had previously failed to respond successfully to IFN-based therapy. This report describes the rationale for the design of a Phase II trial of a botanical product in treating previous nonresponders, the challenges encountered in study design and implementation, and presents baseline characteristics of the enrolled cohort.

## Methods

### Selection of trial participants

This clinical trial studying silymarin treatment in chronic hepatitis C included treatment-resistant patients. Several studies estimated that up to 86% of patients are deferred from conventional IFN treatment due to medical or psychiatric comorbidities [6–8]. Adverse events are common in patients with chronic hepatitis C treated with combination of pegylated IFN and ribavirin [23]. The impressive therapeutic response rates from Phase III clinical trials of pegylated IFN and ribavirin reflect a highly select patient population, that is, those who are deemed to be ideal treatment candidates and who have no comorbidities that would hamper their ability to tolerate a rigorous therapy. The remainder of patients with chronic hepatitis C, those with absolute or relative contraindications and those who did not achieve sustained virological response with previous therapy, could potentially benefit from other therapies.

Thus, as conventional treatment options existed for chronic hepatitis C when this trial began and currently, this study focused on the subset who had failed to have a sustained virological response to previous IFN-based therapy (treatment resistant), then the standard of care, rather than treatment-naïve patients. Lack of a sustained virological response was defined as detectable virus less than 6 months following end of treatment:

1. HCV RNA remained detectable throughout the therapy (nonresponders).
2. HCV RNA dropped to below detectable levels during treatment but became detectable while still on therapy (breakthrough).
3. HCV RNA dropped to below detectable levels during treatment but became detectable once therapy was discontinued (relapsers).

After they completed screening (see Table 1), 154 patients with chronic hepatitis who did not have a sustained virological response to previous IFN-based antiviral therapy, with or without ribavirin, were allocated, using an adaptive minimization randomization scheme, to one of three treatment arms: 420 mg of silymarin (three Legalon^®^ capsules + two placebo capsules), 700 mg of silymarin (five Legalon^®^ capsules), or placebo (five placebo capsules). Thus, five capsules of study treatment were taken orally thrice daily for 24 weeks. At screening, eligible patients were instructed to avoid the use of milk thistle products, and most were randomized within 28 days.

**Table 1.** Enrollment criteria for the Phase II randomized trial of silymarin for chronic hepatitis C

Enrollment criteria
Key inclusion criteria
• Serum HCV RNA above quantifiable level of detection by any assay after the end of previous therapy
• ALT ≥ 65 IU/L (i.e., approximately 1.5 times upper limit of normal) obtained during the screening period
• Previous treatment with any IFN-based therapy without sustained virological response
Key exclusion criteria
• Use of silymarin or other milk thistle preparations within 30 days prior to screening
• Use of other antioxidants such as vitamin E, vitamin C, glutathione, alpha-tocopherol, or nonprescribed complementary alternative medications (including dietary supplements, megadose vitamins, herbal preparations, and special teas) within 30 days prior to screening. A multivitamin at standard doses will be allowed
• Use of silymarin or other antioxidants or nonprescribed complementary alternative medications (as above) during the screening period or patient unwilling to refrain from taking these medications through completion of the study
• Any antiviral therapy within 6 months prior to screening visit
• Known allergy/sensitivity to milk thistle or its preparations
• Evidence of poorly controlled diabetes (defined as hemoglobin A1c > 8% in patients with diabetes)
• Lactose intolerance defined as patient reported inability to tolerate milk products
• Previous liver biopsy that demonstrated the presence of moderate to severe steatosis or evidence of steatohepatitis
• Positive test for anti-human immunodeficiency virus or hepatitis B surface antigen within 5 years of screening
• Average alcohol consumption of more than one drink or equivalent (>12 g) per day or more than two drinks on any 1 day over the 30 days prior to screening. Patients who met either criterion more than 30 days ago must have consumed a monthly average of 12 g or less per day of alcohol for at least 6 months prior to screening
• Serum creatinine level 2.0 mg/dL or greater at screening or creatinine clearance ≤ 60 cc/min or currently on dialysis. The creatinine clearance will be calculated according to Cockcroft–Gault
• Evidence of drug abuse within 6 months prior to screening or during the screening period
• Evidence of decompensated liver disease defined as any of the following: serum albumin < 3.2 g/dL, total bilirubin > 1.5 mg/dL, or protime international normalized ratio > 1.3 times normal at screening, or history or presence of ascites or encephalopathy, or bleeding from esophageal varices
• Participation in a research drug trial, exclusive of the SyNCH Phase I trial, within 6 months of enrollment

**Figure 2.**
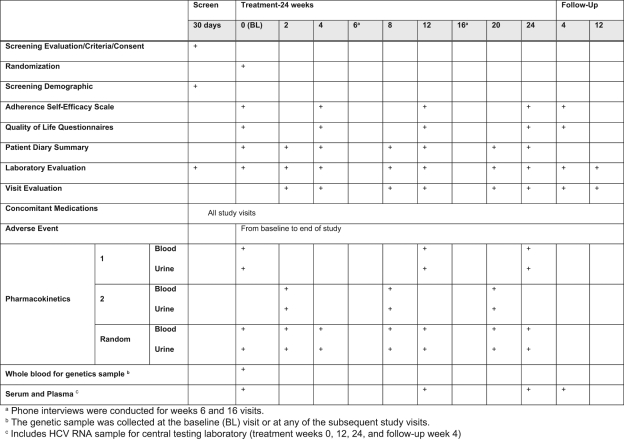
Study visits for the Phase II trial.

Patients with any of the HCV genotypes were eligible, provided they met the treatment-resistant definition. Study visits were planned as outlined in Figure 2; they were planned to provide the opportunity to perform population-based pharmacokinetic studies. In addition, the multiple study visits and trial infrastructure provided the opportunity to collect serum samples from a well-characterized cohort that can be assayed for mechanistic studies, including exploring immunologic effects of chronic silymarin exposure and biomarker screening.

An exemption subcommittee of the overall steering committee, comprising two hepatologists from the NIH/NIDDK who did not enroll participants, adjudicated whether participants who did not meet all inclusion, or who met at least one exclusion, criterion could be enrolled in the trial. During accrual, 25 exemptions were requested and 24 were approved. None of the exemptions were related to alanine aminotransferase (ALT), which was the basis of the primary efficacy end point of the trial.

## Phase II trial objectives and outcome variables

The primary objectives of the Phase II trial are to assess the safety profile of silymarin versus placebo across three exposure levels (0, 420, and 700 mg) and to evaluate whether a 24-week course of silymarin has a discernible impact on ALT, a biochemical marker of hepatic disease. The primary outcome variable for efficacy is serum ALT level of 45 IU/L (approximate maximum of normal range) or less, or a decrease in ALT from baseline level by at least 50% to less than 65 IU/L (approximately 1.5 times the upper limit of normal) at the end of the 24-week treatment period. The primary outcome variable for safety is the occurrence of a dose-limiting toxicity during the 24-week treatment period.

Secondary objectives of the trial include assessing changes in serum levels of HCV RNA during silymarin therapy, characterizing the population pharmacokinetics and pharmacodynamics of silymarin isomers, including silibinins A and B, isosilibinins A and B, silicristin, and silidianin, following administration of silymarin to participants with HCV, and exploring relationships between silymarin therapy and clinical markers of HCV hepatic disease activity. The relationship between dose and change in log_10_ (ALT) will also be assessed.

## Choice of primary outcome variable

Perhaps the most direct measure of improvement in liver disease is obtained by assessing the grade of necroinflammatory activity and stage of fibrosis in paired liver biopsies obtained before and after therapy [24]. Indeed, most therapeutic trials of liver disease have focused on liver biopsy end points. However, liver biopsies confer some risks and discomfort to the patient and are costly to perform. More importantly, it is possible that significant changes in hepatic histology cannot be discerned in trials conducted for relatively short treatment periods (i.e., weeks to months). These factors, as well as the inherent variability of liver biopsy sampling and its histologic scoring, compelled the investigators to develop a nonbiopsy primary outcome variable for this Phase II trial.

Therefore, when designing this trial for resistant disease, the approach focused on a clinically important end point (changes in ALT level), which did not require a liver biopsy. Precedence for such this choice of outcome was available from earlier trials of antiviral therapies for chronic hepatitis C, prior to the advent of qualitative assays for HCV RNA, which utilized biochemical response (changes in hepatic enzyme activity) as the primary end point [25–29]. Histologic improvement, however, frequently accompanied biochemical improvement in these studies during IFN therapy, even when sustained virological response was not observed. Many hepatologists consider that serum ALT activity may provide an indirect marker for improvement in disease activity, although this supposition has not been demonstrated conclusively [3,4,29–31].

Thus, the primary outcome assessment for this 24-week Phase II study of silymarin focuses on quantifying changes in ALT levels to explore initial evidence for efficacy of silymarin in treatment-resistant chronic HCV infection. The primary end point of the trial incorporates stringent criteria for changes in ALT levels. In order to detect such marked changes in ALT, the enrollment criteria also required a high threshold for inclusion (at least 65 IU/L). It is likely that therapeutic agents that could improve surrogate biochemical markers of disease activity and, perhaps, ameliorate complications of hepatic disease even without affecting HCV RNA, would be an attractive adjunct to currently available medications.

## Rationale for revising eligibility criteria to include patients with well-compensated cirrhosis

At initial study implementation, trial eligibility was restricted to patients without cirrhosis noted on any previous liver biopsy. Subsequently, the protocol was amended to permit enrollment of patients with well-compensated cirrhosis. This protocol change resulted primarily from two considerations. The first consideration was that a significant proportion of patients with chronic HCV have cirrhosis but were excluded from the trial. During the trial planning phase, there was considerable interest in including patients with well-compensated cirrhosis as they are a significant subgroup of those with chronic disease. But due to insufficient product safety data in the patient population, they were excluded. Another consideration was the lower than anticipated enrollment rate across all sites during the trial enrollment phase, in part due to excluding a significant fraction of patients with chronic HCV infection and cirrhosis, a factor associated with nonresponse to IFN-based therapies.

The Phase I trial had provided safety data across many dose levels among HCV patients without documented cirrhosis. After the Phase II trial was initiated, however, pharmacokinetic and safety data from a newly published study suggested that cirrhotic patients could be treated safely with an intravenous form of silibinin (Legalon^®^ SIL). It was noted that circulating levels associated with the intravenous form of the product were considerably higher than those associated with orally administered silymarin [12]. In that study, higher serum levels of silibinins A and B were achieved during daily intravenous infusions for a 2-week period with no dose-related adverse effects; patients with cirrhosis who had failed prior therapy were an important subgroup of study participants [12].

However, the safety profile observed in patients with cirrhosis with the intravenous form of silibinin (Legalon^®^ SIL) may not be identical to silymarin since they are chemically two different drugs. Silymarin is composed of six flavonolignans including silibinins A and B, whereas Legalon^®^ SIL is composed of water-soluble hemisuccinate covalent esters of only silibinins A and B. Therefore, once the protocol was modified to include those with well-compensated cirrhosis, after approval from the DSMB and relevant institutional review boards (IRBs), the study adopted a more stringent plan to monitor safety in trial participants with well-compensated cirrhosis. This plan included monitoring plasma concentrations in the event of a serious adverse event or hepatic biochemical test elevation in someone with cirrhosis, since the only oral silymarin pharmacokinetic data published to date indicated that blood levels were likely to be substantially higher in cirrhotics than in noncirrhotics [32].

## Sample size considerations

The sample size for this study was estimated based on the primary comparison of the proportions of participants in each arm who attain the primary end point, as defined earlier. The total sample size of 153 was calculated to provide 80% power to detect a clinically meaningful difference in outcome between the placebo and silymarin arms if there was no dose–response relationship, and over 90% if there was a dose–response relationship. This sample size calculation assumed a spontaneous ALT normalization (primary end point) in 15% of placebo-treated participants and assumed a clinically significant (25%) improvement in the success rate for the silymarin-treated group. A total of 154 participants were enrolled.

## Baseline characteristics of participants in the Phase II trial

The baseline characteristics of the participants enrolled in the Phase II trial (summarized in Table 2) are representative of the population of nonresponders to current hepatitis C therapy of pegylated IFN and ribavirin [33]. The median age of the predominantly male (71.4%) participants was 54 years. As anticipated, many of these previous IFN-based therapy nonresponders had characteristics of difficult-to-successfully-treat patients including genotype 1 infection (91.4%), cirrhosis (27.9%), overweight or obese (85.0%), and elevated fasting blood glucose of at least 100 mg/dL (36.6%) [34] (Table 2). The final cohort is composed of 20% African-American participants, similar to the percentage enrolled in nonresponder trials but higher than the percentage enrolled in clinical trials involving treatment-naïve patients [3–5]. Nearly 90% of the cohort reported regular use of medications (prescription or over-the-counter preparations) at study enrollment, with almost 30% reporting taking one or more alternative or complementary medications. Dietary supplements reported by 28% of trial participants at enrollment included a variety of products such as nutrients and botanicals.

**Table 2.** Baseline characteristics of the Phase II trial cohort (*N* = 154)

Patient characteristics	
Men, *n* (%)	110 (71.4)
Race, *n* (%)	
White or Caucasian	114 (75.0)
Black or African-American	31 (20.4)
Other	9 (4.6)
Age (years)	
Median (25th percentile, 75th percentile)	54 (51, 58)
Minimum, maximum	31, 73
Weight (kg) – male	
Mean (SD)	92.2(15.3)
Minimum, maximum	62.5, 150.8
Weight (kg) – female	
Mean (SD)	82.6 (18.8)
Minimum, maximum	47.3, 116.4
BMI (kg/m^2^) – male	
Median (25th percentile, 75th percentile)	29.1 (26.6, 32.0)
Minimum, maximum	18.0, 48.5
Missing	1
BMI (kg/m^2^) – female	
Median (25th percentile, 75th percentile)	30.0 (25.9, 34.4)
Minimum, maximum	19.0, 43.1
Well-compensated cirrhosis, *n* (%)	43 (27.9)
Diabetics, *n* (%)	21 (13.6)
ALT (IU/L)	
Median (25th percentile, 75th percentile)	107 (83, 150)
Minimum, maximum	60, 553
Total bilirubin (mg/dL)	
Median (25th percentile, 75th percentile)	0.8 (0.6, 1.0)
Minimum, maximum	0.2, 1.9
HCV genotype, *n* (%)	
1a	54 (35.5)
1b	21 (13.8)
1 not otherwise specified	64 (42.1)
2b	1 (0.7)
2 not otherwise specified	4 (2.6)
3a	1 (0.7)
3 not otherwise specified	5 (3.3)
4a	2 (1.3)
Missing	2
HCV RNA quantitative (log_10_)	
Median (25th percentile, 75th percentile)	6.2 (5.8, 6.6)
Minimum, maximum	3.8, 7.7
Missing	2
Fasting glucose (mg/dL), *n* (%)	
<100 mg/dL	90 (63.4)
≥100 mg/dL	52 (36.6)
Missing	12
Concomitant medications, *n* (%)	
No medications at baseline	20 (13.0)
Anti-inflammatory and analgesic medications	52 (33.8)
Statins and related drugs	6 (3.9)
Vitamins/herbals/minerals	43 (27.9)
Diabetes medications	13 (8.4)
Antidepressants	41 (26.6)
Other	116 (75.3)

## Discussion

Despite a large body of literature describing the use of silymarin over several decades, the published evidence of clinical efficacy in hepatic disease is equivocal. Thus, it was important to systematically study the potential benefit of silymarin in a common chronic liver disease. The large disease burden, with lack of effective therapies, along with frequent poor tolerability of IFN and ribavirin therapy by chronic hepatitis C patients made them an important group of patients in which to evaluate silymarin. The previous lack of consistency across the few studies with regard to efficacy end points, silymarin preparations, and liver disease diagnosis was further impetus to implement a phased development plan for clinical testing. During the early planning stages, it was apparent that, as for most botanical products, dose-finding pharmacokinetic studies were sparse and doses of different preparations had been identified arbitrarily for assessment in liver diseases. In addition, there were no preexisting studies of botanical-food or botanical-drug pharmacokinetics. It was also important to identify an industry collaborator motivated to participate in the development plan, which would provide study product of standard composition and which also could provide a product that complied with FDA IND requirements for clinical testing. Implementing a phased approach, the investigators initially performed a dose-ranging Phase I study to assess pharmacokinetics and safety, after which two doses of 420 and 700 mg taken three times a day were targeted for evaluation in the Phase II study.

The cohort reflects the treatment-resistant HCV population in the United States in many ways, as it is predominantly male, with a number of clinical parameters associated with resistant disease, including genotype 1, obesity, diabetes, and cirrhosis [3–5, 34,35]. Several reports have suggested that African-Americans do not respond well to hepatitis C therapy [36]. Thus, it was anticipated that the cohort would consist of substantial numbers of African-Americans. Although 20% of the cohort is African-American, it is lower than might be expected in a cohort of treatment-resistant HCV patients. This is likely due to several factors, including the typically lower ALT levels observed in African-American patients, compared with Caucasian with chronic HCV, and the stringent inclusion threshold of at least 65 IU/L for the current trial [37]. In addition, data regarding botanical product use from the 2007 NHIS CAM survey indicate much greater use for such products in Caucasians (43%) than African-Americans (26%), suggesting that Caucasians might have greater interest in enrolling in studies of botanical therapies [14].

While IFN and ribavirin are known to cause significant side effects, silymarin has been well tolerated by patients with chronic hepatitis C [9–12]. As frequently noted among patients with serious chronic illnesses, quality of life is significantly diminished in patients with chronic hepatitis C. This aspect of the disease is associated with considerable work absence due to functional disability [38]. Given the side effects of conventional HCV therapy, many patients are reluctant to adhere to standard treatment regimens. Many patients explore other potential therapies, such as silymarin, to complement conventional therapies, or less frequently, as an alternative to conventional therapies. In view of the fairly widespread use of silymarin, the current phased investigation of its use in chronic hepatitis C will provide a rigorous assessment of its safety and associated quality of life, as well as further pharmacokinetic data on silymarin in patients with hepatic disease.

## Summary

The phased-study approach undertaken for silymarin as described in this report addressed several unique challenges to design of clinical trials programs of botanical products. As products may be marketed in the United States without standard chemistry data provided and usually with limited data on pharmacokinetics and dosing, much early-phase testing is required before clinical trials can be designed and implemented. Unlike studies of conventional pharmaceuticals, early work to identify a manufacturer and a standard botanical formulation must be undertaken to begin the process of preliminary preclinical assessment. Verifying the acceptability of testing for IND filings with the FDA is an important early step in planning clinical testing of all new products for specific disease indications but are particularly challenging for botanical products due to lack of data, manufacturing requirements, and standard product composition. The phased-study approach implemented by the SyNCH Study Group for silymarin has required several years of effort but has been successful in providing important chemistry, pharmacokinetic, and dosing data on a commonly used botanical product. Enrollment in the trial began in May 2008 and concluded in May 2010. The treatment and follow-up phases of the trial were completed by March 2011.

## Appendix

### The SyNCH HCV study group

#### Clinical centers

Beth Israel Deaconess Medical Center, Boston, MA – Nezam Afdhal, MD; Joseph Colagreco, MS, NP Thomas Jefferson University, Philadelphia, PA – Divya Gupta, MD, MS; Cynthia Miller, RN; Victor Navarro, MD; Manisha Verma, MD University of North Carolina, Chapel Hill, NC – Eric Borg, PharmD; Paris Davis, BA; Michael Fried, MD; Roy Hawke, PharmD, PhD; Meredith Howell, PharmD; Tedi Soule, PharmD University of Pennsylvania, Philadelphia, PA – David E. Kaplan, MD; Christine Kennedy, Amy Micheli, MPH; K. Rajender Reddy, MD; Amina Wirjosemito, MPH.

#### Data-coordinating center

University of Pittsburgh, Pittsburgh, PA – Steven Belle, PhD, MScHyg; Joy Bowen, BA; Marcia Kurs-Lasky, MS; Sharon Lawlor, MBA; Abdus Wahed, PhD; Ella Zadorozny, MS.

#### National Institutes of Health

National Center for Complementary and Alternative Medicine – Linda Duffy, PhD; Catherine Meyers, MD NIDDK – Edward Doo, MD, Jay Hoofnagle, MD, Leonard Seeff, MD.

#### Industry partners

Rottapharm | Madaus (Monza, Italy and Cologne, Germany) provided study medication and placebo for the trial – Massimo D’Amato, MD, Lucio Rovati, MD.
